# Diagnostic and Therapeutic Implications of Organic Delusional Disorder due to Tuberculous Adrenalitis

**DOI:** 10.1155/2022/5056976

**Published:** 2022-06-01

**Authors:** Pooja Govind, Karthick Subramanian, Suriya Kumar

**Affiliations:** Department of Psychiatry, Mahatma Gandhi Medical College & Research Institute (MGMC&RI), Sri Balaji Vidyapeeth (Deemed to be University), Pondicherry, India

## Abstract

Primary adrenal insufficiency rarely occurs due to infections, which consequently involves destruction or dysfunction of both adrenal cortices. Tuberculous adrenalitis is still a frequent cause of adrenal insufficiency in developing countries. We present the case of origin of multiple delusions along with recurrent spells of vomiting and giddiness in a patient with pulmonary tuberculosis (TB). A thorough medical history combined with a panel of biochemical and endocrine investigations revealed disseminated TB with choroid tubercles and adrenal infiltration leading to primary adrenal insufficiency. A diagnosis of organic delusional disorder secondary to disseminated TB-associated adrenal insufficiency was considered. The patient was managed with risperidone and antitubercular drugs. The psychosis improved and the patient was stable during the follow-up. The present case report adds to the literature on diagnostic challenges associated with psychosis due to adrenal insufficiency secondary to disseminated TB.

## 1. Introduction

Around 26% of global cases of tuberculosis (TB) are present in India [1]. Psychiatric comorbidities like depression, anxiety, substance abuse, and psychosis in TB lead to significant physical and social disability [2, 3]. Psychosis in TB presents with abrupt or acute onset of hallucinations, delusions, and behavioral problems [4]. Apart from drug-related causes, complications of TB including meningitis and adrenalitis can induce psychosis in TB [1, 5].

We present the diagnostic and therapeutic implications of adrenal insufficiency due to tubercular adrenalitis in inducing psychosis.

## 2. Case Description

A 56-year-old male, with irritability, aggression, suspiciousness, and sleep disturbances for 1.5 years, presented to psychiatric emergency services after an acute exacerbation. He had a history of irregularly treated type 2 diabetes mellitus and hypothyroidism. Two years earlier, the patient had developed low-grade fever, cough, and weight loss and then diagnosed as pulmonary TB. After three months of anti-TB treatment (ATT), he developed episodes of altered sensorium and generalised tonic-clonic seizures secondary to hyponatremia and normal pressure hydrocephalus, which resolved with conservative treatment (Table 1). One month later, the patient had recurrent dizziness, nausea, and vomiting. Evaluations revealed episodic hyponatremia and reduced serum cortisol. The patient could not afford further investigations. Considering pulmonary TB and physical signs of adrenal insufficiency, tubercular adrenalitis was provisionally diagnosed and empirical steroid treatment was initiated. Poor compliance to steroids resulted in a relapse of adrenal sufficiency along with an acute-onset, continuous course of psychiatric illness characterized by irritability, suspiciousness, and biological and sociooccupational dysfunction leading to the current clinical presentation. His premorbid, past, family, and substance use history were nil contributory. Mental status examination revealed delusions of reference, persecution, and misidentification. The patient was diagnosed with organic delusional (Schizophrenia-like) disorder (F06.2) as per the International Classification of Diseases–10^th^ edition (ICD-10) [6].

Psychosis improved with risperidone (2 to 6 mg/day) (Brief Psychiatric Rating Scale scores: baseline-58 and follow-up-32) over 2-3 months. His adrenal insufficiency was symptomatically managed with an endocrinologist liaison and reinitiation of steroid therapy. Adherence was ensured through telepsychiatry services. Written informed consent was obtained from the patient and caregiver prior to the write-up of this report.

## 3. Discussion

The present case highlights the incidence of organic delusional disorder secondary to adrenal insufficiency in a patient with TB.

Most common cause of adrenal insufficiency is autoimmune followed by infections. A significant proportion of patients with Addison's disease develop psychiatric symptoms including frank psychosis [2, 7, 8]. Our findings concur with a recent case study which found that psychosis can be the primary and predominant manifestation of adrenal insufficiency even in the absence of past or family history of psychiatric illness [9]. The pathophysiology of psychosis in adrenal insufficiency involves hyponatremia, hypoglycemia, decreased glucocorticoids, and metabolic abnormalities among various others [10]. Recent reports reveal that proinflammatory cytokines consequent to decreased cortisol may underlie the onset of psychosis in adrenal insufficiency [11].

The main limitation of the present case study is the unavailability of further etiopathological explorations, especially hormonal and neuroimaging investigations, because of logistic difficulties in a resource-limited setting. However, systematic and thorough clinical evaluation of adrenal insufficiency and psychosis aided in early clinical recovery in this patient.

Thus, the present report adds to the existing literature that (i) delusion of misidentification can occur in psychosis secondary to adrenal insufficiency and (ii) careful evaluation of clinical signs of adrenal insufficiency and correlation of onset of psychosis will aid in timely diagnosis and intervention resulting in early clinical recovery, especially in resource-limited settings.

## Figures and Tables

**Table 1:** Blood biochemistry and imaging findings.

Investigation	Period of evaluation from onset of PTB	Value
Chest X-ray	0 month	L–mid zone fibrosis
Bronchoscopy and GeneXpert	0 month	Positive for MTB
CT brain	3^rd^ month	Normal pressure hydrocephalus, dilated ventricles, and cerebral atrophy
MRI brain	3^rd^ month	Diffuse cerebral atrophy and ex vacuo dilatation of ventricles
Sodium	3^rd^ month	118 meq/L
Fundoscopy	3^rd^ month	Choroid tubercles
Cortisol (7 am sample)	4^th^ month	2.25 mcg/dL
Hemoglobin	Current admission	8.3 gm/dL
TSH	Current admission	58.60 mIU/L
RBS	Current admission	176 mg/dL
HbA1c	Current admission	12.6

CT: computed tomography; HbA1c: hemoglobin A1c; MRI: magnetic resonance imaging; MTB: mycobacterium tuberculosis; RBS: random blood sugar; PTB: pulmonary tuberculosis; TSH: thyroid-stimulating hormone.
